# Cardiovascular Risk with Non-Steroidal Anti-Inflammatory Drugs: Systematic Review of Population-Based Controlled Observational Studies

**DOI:** 10.1371/journal.pmed.1001098

**Published:** 2011-09-27

**Authors:** Patricia McGettigan, David Henry

**Affiliations:** 1 Hull York Medical School, Hull, United Kingdom; 2 Institute for Clinical Evaluative Sciences, Toronto, Ontario, Canada; 3 Department of Medicine, University of Toronto, Toronto, Ontario, Canada; 4 Discipline of Clinical Pharmacology, School of Medicine and Public Health, University of Newcastle, Newcastle, Australia; University of Pennsylvania School of Medicine, United States of America

## Abstract

**Background:**

Randomised trials have highlighted the cardiovascular risks of non-steroidal anti-inflammatory drugs (NSAIDs) in high doses and sometimes atypical settings. Here, we provide estimates of the comparative risks with individual NSAIDs at typical doses in community settings.

**Methods and Findings:**

We performed a systematic review of community-based controlled observational studies. We conducted comprehensive literature searches, extracted adjusted relative risk (RR) estimates, and pooled the estimates for major cardiovascular events associated with use of individual NSAIDs, in different doses, and in populations with low and high background risks of cardiovascular events. We also compared individual drugs in pair-wise (within study) analyses, generating ratios of RRs (RRRs). Thirty case-control studies included 184,946 cardiovascular events, and 21 cohort studies described outcomes in >2.7 million exposed individuals. Of the extensively studied drugs (ten or more studies), the highest overall risks were seen with rofecoxib, 1.45 (95% CI 1.33, 1.59), and diclofenac, 1.40 (1.27, 1.55), and the lowest with ibuprofen, 1.18 (1.11, 1.25), and naproxen, 1.09 (1.02, 1.16). In a sub-set of studies, risk was elevated with low doses of rofecoxib, 1.37 (1.20, 1.57), celecoxib, 1.26 (1.09, 1.47), and diclofenac, 1.22 (1.12, 1.33), and rose in each case with higher doses. Ibuprofen risk was seen only with higher doses. Naproxen was risk-neutral at all doses. Of the less studied drugs etoricoxib, 2.05 (1.45, 2.88), etodolac, 1.55 (1.28, 1.87), and indomethacin, 1.30 (1.19, 1.41), had the highest risks. In pair-wise comparisons, etoricoxib had a higher RR than ibuprofen, RRR = 1.68 (99% CI 1.14, 2.49), and naproxen, RRR = 1.75 (1.16, 2.64); etodolac was not significantly different from naproxen and ibuprofen. Naproxen had a significantly lower risk than ibuprofen, RRR = 0.92 (0.87, 0.99). RR estimates were constant with different background risks for cardiovascular disease and rose early in the course of treatment.

**Conclusions:**

This review suggests that among widely used NSAIDs, naproxen and low-dose ibuprofen are least likely to increase cardiovascular risk. Diclofenac in doses available without prescription elevates risk. The data for etoricoxib were sparse, but in pair-wise comparisons this drug had a significantly higher RR than naproxen or ibuprofen. Indomethacin is an older, rather toxic drug, and the evidence on cardiovascular risk casts doubt on its continued clinical use.

*Please see later in the article for the Editors' Summary*

## Abstract 2

David Henry and colleagues reevaluate the evidence from observational studies on the cardiovascular risk associated with non-steroidal anti-inflammatory drugs.

## Editors' Summary

**Background:**

The analgesic (pain relieving), anti-pyretic (fever reducing), and anti-inflammatory (inflammation reducing) properties of the class of drug called non-steroidal anti-inflammatory drugs (NSAIDs)—so called to distinguish this class of drug from steroids, which have similar but additional effects—make NSAIDs one of the most frequently used drugs for the symptomatic treatment of many common conditions. Some preparations of NSAIDs can be bought over the counter, and all are available on prescription, but this class of drug has well documented side effects and risks: people taking NSAIDs are on average four times more likely to develop gastrointestinal complications than people not taking these drugs (that is, the relative risk of gastrointestinal complications is 4), and the relative risk for associated cardiovascular complications—cardiovascular events during treatment with NSAIDs has been one of the most studied adverse drug reactions in history—ranges from 1.0 to 2.0.

**Why Was This Study Done?:**

Several large systematic reviews, including one conducted by these researchers, have previously highlighted apparent differences in cardiovascular risk between individual drugs, but these reviews have provided limited information on dose effects and relevant patient characteristics and have not directly compared the cardiovascular risks of each drug. Furthermore, most of these analyses extensively investigated only a few drugs, with little information on some widely available compounds, such as etoricoxib, etodolac, meloxicam, indomethacin, and piroxicam. Therefore, the researchers conducted this study to update cardiovascular risk estimates for all currently available NSAIDs and to compare the risks between individual drugs. In order to investigate the likely effects of over-the-counter use of NSAIDS, the researchers also wanted to include in their review an analysis of the cardiovascular risk at low doses of relevant drugs, over short time periods, and in low risk populations.

**What Did the Researchers Do and Find?:**

The researchers included only controlled observational studies in their literature search and review (conducted by searching a wide range of databases for studies published from 1985 until November 2010) because randomized controlled trials have reported only small numbers of cardiovascular events that are insufficient for the purposes of this study. The researchers assessed the methodological quality of selected studies, analyzed adjustment variables (for example, age, sex, other medications), and summarized overall results for individual drugs across studies as pooled relative risk estimates. For the subsets of studies that provided relevant data, they pooled within-study relative risk estimates with high and low doses and in people at high and low risk of cardiovascular events, and performed a series of within-study (pair-wise) comparisons and for each pair of drugs, to estimate their comparative relative risks by using a validated online tool to give a ratio of relative risks.

Using this methodology, the researchers included 30 case-control studies and 21 cohort studies: the highest overall risks were with rofecoxib and diclofenac, and the lowest risks were with ibuprofen and naproxen, The researchers found that risk was elevated with low doses of rofecoxib, celecoxib, and diclofenac, and rose with higher doses. Ibuprofen risk was only evident with higher doses. Naproxen did not cause any additional risks at any dose. Of the less studied NSAIDs, etoricoxib, etodolac, and indomethacin had the highest risks. In the pair-wise comparisons, the researchers found that etoricoxib had a higher relative risk than ibuprofen and naproxen, etodolac was not significantly different from naproxen and ibuprofen, and naproxen had a significantly lower risk than ibuprofen. Finally, the researchers showed that relative risk estimates were constant with different background risks for cardiovascular disease and increased early the course of treatment.

**What Do These Findings Mean?:**

This updated systematic review gives some new information on some familiar NSAIDs, and provides potentially important information on some little studied ones, which will help to inform clinical and regulatory decisions. The specific findings suggest that among widely used NSAIDs, naproxen and low-dose ibuprofen are least likely to increase cardiovascular risk, whereas diclofenac in doses available without prescription elevates risk. Based on sparse data, etoricoxib has a high risk of cardiovascular events and is similar to drugs that have been withdrawn because of safety concerns. Indomethacin is an older, rather toxic drug, and the new evidence on cardiovascular risk casts doubt on its continued clinical use.

**Additional Information:**

Please access these websites via the online version of this summary at http://dx.doi.org/10.1371/journal.pmed.1001098.

- Wikipedia defines and discusses NSAIDs
- The UK National Health Service and MedicineNet have useful information on NSAIDs that is suitable for patients
- The National Prescribing Service in Australia has a range of information on the use of NSAIDs

## Introduction

The risk of cardiovascular events during treatment with non-steroidal anti-inflammatory drugs (NSAIDs) has been one of the most studied adverse drug reactions in history. Concern was initially evoked by a consideration of basic mechanisms of the drugs [1]. This was reinforced by signals that emerged in large clinical trials designed (primarily) to examine gastrointestinal effects [2],[3]. Pharmaco-epidemiological studies have confirmed and quantified these effects using large linked administrative databases in several countries [4],[5].

In contrast to gastrointestinal complications, where the average relative risk (RR) was estimated to be around 4, many of the RR estimates for cardiovascular complications have been in the range of 1.0 to 2.0 [6],[7]. This limits the statistical power to examine variations in risk within individual studies. But it is important to know whether risk varies with individual drugs, with dose and duration of therapy, or according to patient characteristics, such as underlying cardiovascular risk. There are additional concerns about the risk associated with non-prescription NSAIDs that are available in low-dose forms, including ibuprofen, naproxen, and diclofenac [8].

Exploration of these factors requires systematic review and meta-analysis of the available studies. Several large systematic reviews have been published covering randomised trials and non-randomised pharmaco-epidemiological studies [7],[9]–[12]. These reviews have highlighted apparent differences between individual drugs, but have provided limited information on dose effects and relevant patient characteristics, and have not provided direct comparisons between drugs on the basis of cardiovascular risk. Most of the analyses have involved a few extensively investigated drugs, with little information on some widely available compounds, such as etoricoxib, etodolac, meloxicam, indomethacin and piroxicam.

Our purpose here is to update our previously published review of large observational studies in order to provide updated risk estimates for the most widely studied drugs, those that have been less commonly investigated, and newer drugs that were not included in our previous systematic review [7]. We wanted to obtain comparative estimates of risk for individual drugs and to investigate the likely effects of non-prescription use through examination of risk at low doses of relevant drugs, over short time periods and in low risk populations.

## Methods

### Overview

We did not develop a new protocol as we followed closely the methods outlined in an earlier version of this work [7]. We confined our analysis to non-randomised controlled observational designs. Placebo-controlled trials of NSAIDs have captured fairly small numbers of cardiovascular events, which are insufficient to inform all of the discriminatory analyses we proposed here. In addition, the available randomised data have been summarised in recent and on-going meta-analyses [9],[10],[13].

We used a range of complementary approaches to analyse the data. The overall results for individual drugs were summarised across studies as pooled RR estimates with 95% confidence intervals (CIs). The numbers of studies contributing to these estimates varied with individual drugs. For the sub-sets of studies that provided relevant data, we pooled within-study RR estimates with high and low doses and in patients at high and low risk of cardiovascular events. These were pre-specified analyses, and we report the 95% CIs. To compare individual drugs we carried out pair-wise within-study analyses. Because of the large number of possible comparisons, we calculated 99% CIs around these estimates (see below).

### Literature Search and Study Eligibility

Studies were eligible for inclusion if they were controlled (case-control, case-crossover, or controlled cohort designs) and reported on cardiovascular risks associated with the current use of the individual drugs in population settings, with non-use or remote use as the reference exposure. A librarian, an experienced Cochrane reviewer, and one of the authors searched electronic databases for articles published during the period 1 January 1985 to 30 November 2010. The databases included Medline, Embase, PubMed, Cochrane Library, Google Scholar, epidemiological research websites, abstracts of scientific meetings, and bibliographies of relevant studies. The search terms included the generic names of individual drugs, therapeutic classes and modes of action, cardiovascular and cerebro-vascular outcome terms, and study design descriptors (Text S1). We also performed searches using the names of authors known to have conducted research on cardiovascular and cerebro-vascular risks associated with NSAID use. We applied no language restrictions. Titles and abstracts of papers identified by the searches were reviewed by the authors. Searches were re-run using additional search terms identified from papers considered relevant to the review.

### Study Exposures and Outcomes

We wished to study exposure to any NSAID, including selective Cox-2 inhibitors. For the studies included in this analysis, the NSAID prescription was regarded as being current if it covered a period that included the index day or continued to within 1 week or less of the index day (i.e., the day the adverse cardiac event occurred). The most commonly reported outcome was acute myocardial infarction; many studies included coronary heart disease–related death and some reported a composite of myocardial infarction and coronary heart disease death; a minority reported on stroke only. Where it was possible to extract risk estimates for all cardiovascular and cerebro-vascular events separately, we did so.

### Risk of Bias in Individual Studies

The most common adjustment variables used by authors were: age, sex, vascular risk factors, co-morbidities, and prescribed medications. These were handled fairly consistently across the studies. Important factors that were generally not reported as being adjusted for included non-prescription use of aspirin and NSAIDs, smoking, alcohol use, and body mass index. Quality assessment and data extraction were performed in duplicate, with resolution of any discrepancies by consensus. Methodological quality was assessed using the Newcastle–Ottawa Scale [14]. Authors contacted to obtain additional details provided the information requested. Nearly all the studies were conducted using linkage of large electronic health administration databases or electronic health records (Table S1). As these have wide community coverage and document, in real time, data on drug prescribing or dispensing, and subsequent clinical events, they minimise selection biases and some measurement biases that affect classic retrospective case-control designs. Therefore, we felt it appropriate to combine data from different study designs in order to improve our ability to discriminate between individual drugs and drug doses.

### Statistical Analysis

We did not use raw data in the calculations. We extracted the adjusted risk estimates for individual drugs and for the doses reported. Where there were several publications that used the same or overlapping datasets, we extracted the most complete information on cardiovascular risk associated with different doses of individual NSAIDs. We pooled the odds, risk, or hazard ratio estimates for all unique cardiovascular outcomes that represented the most recent use of a NSAID and had been adjusted for potential confounders. We extracted point estimates and 95% CIs in duplicate from each study and combined them using a random effects model for all the comparisons reported here. We performed all statistical analyses in Stats Direct (version 2.7.8). Forest plots were generated using Review Manager (version 5).

Heterogeneity was assessed by the Cochran *Q* and *I* ^2^ statistics. Our purpose in this study was to explain heterogeneity in terms of factors that were associated with variations in RR, including individual drugs, dose, background risk of cardiovascular events, and timing of risk. Our examination of dose effects was restricted to the published dose cut points as we did not have access to individual patient data. Where authors had reported them, we extracted risk estimates for individual drugs measured in populations considered to be at “high” or “low” background risk of cardiovascular events (Table S1). We used the authors' categorisations of risk. The analyses of dose and baseline risk were performed using within-study data. We also extracted risk estimates categorised by duration of exposure, recognising that administrative databases have limited capacity to discriminate time periods of less than one month (the duration of a typical prescription).

We compared paired (within-study) RR values with high and low doses of drugs and in high and low risk populations, and report the heterogeneity statistics as a measure of the statistical significance of any differences. Direct comparisons of overall RR estimates for individual drugs were potentially confounded at study level, so we carried out a series of pair-wise comparisons of drugs that had been included in the same studies. For each pair of drugs, we compared their RRs for a myocardial infarction by the method of Altman and Bland [15], using an online tool [15],[16]. This yielded a ratio of RRs (RRR) with its CI. RRRs were pooled using a random effects model. Because of concerns about multiple testing, we were selective in making comparisons, and we calculated 99%, rather than 95%, CIs around the pooled RRR values. In addition, we chose a threshold *p*-value for reporting based on the Bonferroni adjustment for multiple comparisons (Table 4).

### Selecting Pair-Wise Comparisons of Individual Drugs

In view of the large number of potential pair-wise comparisons, we selected pairs on the basis of the following: (a) the amount of direct comparative data that was available to enable the analyses and (b) the most relevant clinical and regulatory questions. Etoricoxib and etodolac have been little studied. Etoricoxib is not marketed in North America but is widely available elsewhere [17]. Meloxicam is widely used in Australia, where it partially replaced rofecoxib after its withdrawal [18]. Indomethacin is an older drug that is still used in the acute treatment of gout [19]. Diclofenac has been highlighted repeatedly as a cardiovascular risk but has not been compared directly with other commonly used drugs, particularly ibuprofen and naproxen, which, like diclofenac, are available in some countries without prescription [7],[8]. We compared three popular drugs, naproxen, celecoxib, and ibuprofen, as these have emerged from most of the reviews as having lower than average risk, and we wanted to know which was the safest.

### Sensitivity Analyses

Because a number of the pooled RR values were close to one, we carried out sensitivity analyses to determine the strength of an association between cardiovascular events and a hypothetical unmeasured confounder that would be capable of generating the observed RR, if the true value of the association of interest was one. We used a method proposed by Schneeweiss [20]. In calculating these values, we assumed a 15% higher prevalence of the theoretical confounder in the exposed than in the non-exposed population. Because of the large number of analyses conducted here, we limited our sensitivity analyses to the pair-wise comparisons of the drugs, as these are the most important measures of relative harm.

## Results

**Figure 1:**
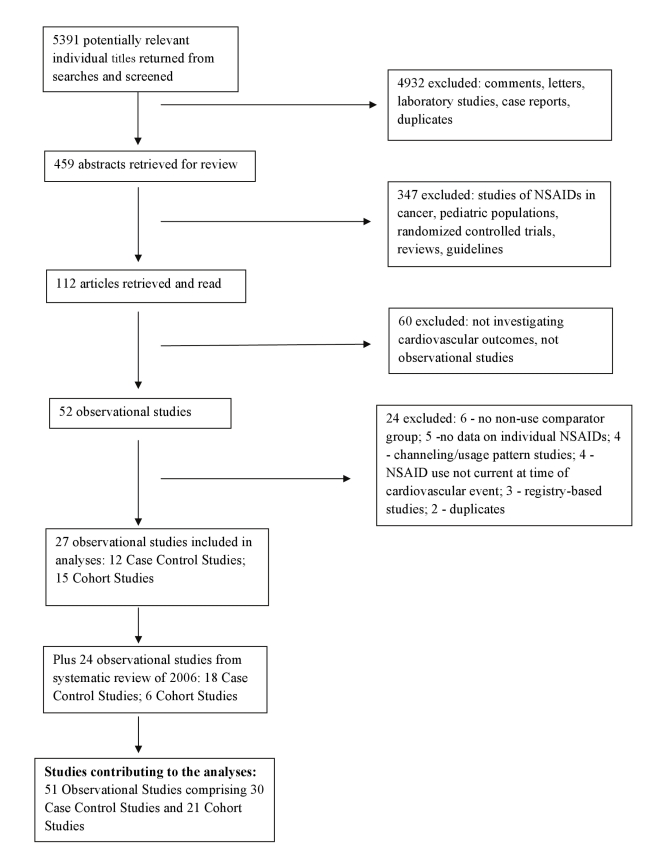
Flow diagram for derivation of studies included in the analyses.

The derivation of the database is described in Figure 1. Lists of included and excluded studies are provided in Text S2. Details of the included studies, including the characteristics of participants, and the analytical and adjustment techniques used by study authors are given in Table S1. The updated database includes 51 studies and 43 unique datasets. Thirty reports of case-control studies included 184,946 cardiovascular events, and 21 cohort studies described outcomes in over 2.7 million exposed individuals. This update more than doubled the amount of statistical information that was included in previously published systematic reviews of pharmaco-epidemiological studies [7],[12].

We assessed the risk of bias of individual studies using the Newcastle–Ottawa Scale [14]. Fully reported case-control studies scored well, 7–8 points from a possible total of 9 points, and cohort studies scored 7–8 points from a possible total of 10 points. There was an insufficient range of scores to assess the relationship between quality and outcome.

**Figure 2:**
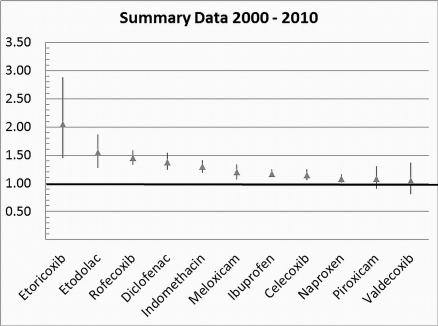
Summary analyses for individual drugs. Vertical axis indicates pooled RR.

**Figure 3:**
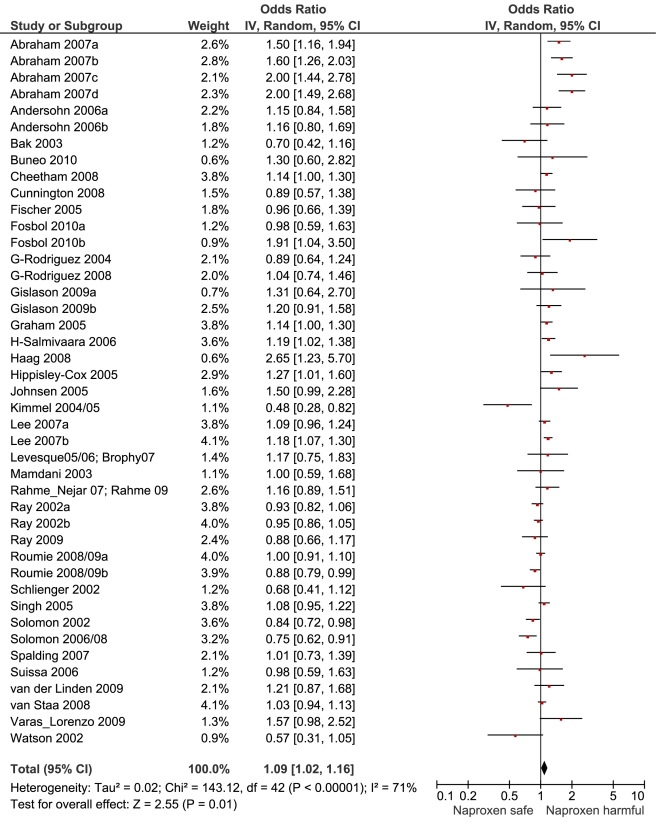
Forest plot for naproxen. **Key for Figures 3-13:** Abraham: 2007a, low risk myocardial infarction; 2007b, average risk myocardial infarction; 2007c, low risk stroke; 2007d, average risk stroke. Andersohn: 2006a, myocardial infarction, cardiovascular death; 2006b, non-fatal stroke. Fosbol: 2010a, myocardial infarction, cardiovascular death; 2010b, fatal, non-fatal stroke. Gislason: 2006a, recurrent myocardial infarction; 2006b, death. Gislason: 2009a, myocardial infarction; 2009b, death. Lee: 2007a, low risk cardiovascular event; 2007b, high risk cardiovascular event. Roumie: 2008/09a, low risk cardiovascular event; 2008/09b, high risk cardiovascular event.

Table 1 highlights the variable numbers of studies and individuals that contributed to the pooled RR estimates for individual drugs. Table 1 and Figure 2 display the summary estimates of RR for each drug, with non-use or remote use as the reference. In Figure 2, the drugs are ranked from highest to lowest RR, based on the point estimates. It is important to note that some of the estimates are imprecise, because of sparse data, as reflected in the wide CIs. Figures 3–13 provide the forest plots from which the summary estimates were derived. The varying numbers of studies included in each estimate make comparisons between drugs difficult because of possible confounding at study level.

**Figure 4 pmed-1001098-g004:**
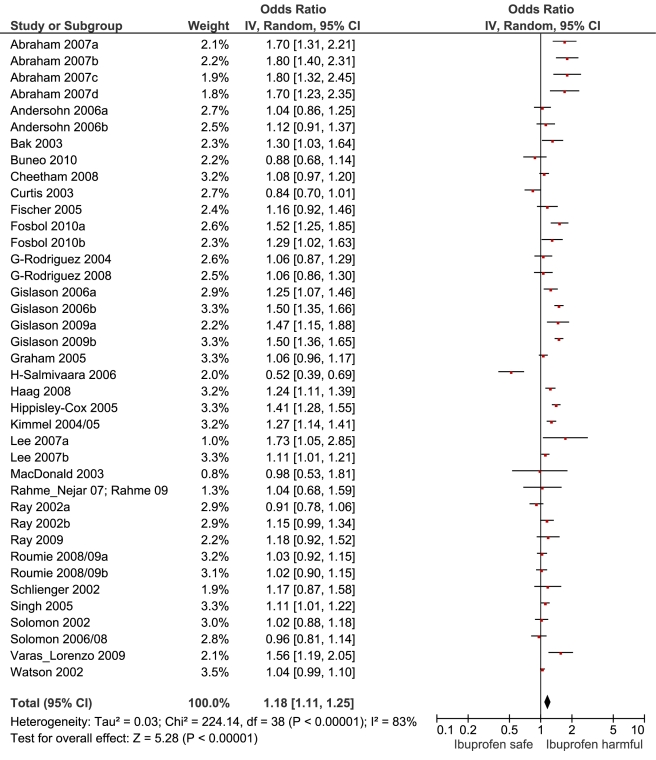
Forest plot for ibuprofen.

**Figure 5 pmed-1001098-g005:**
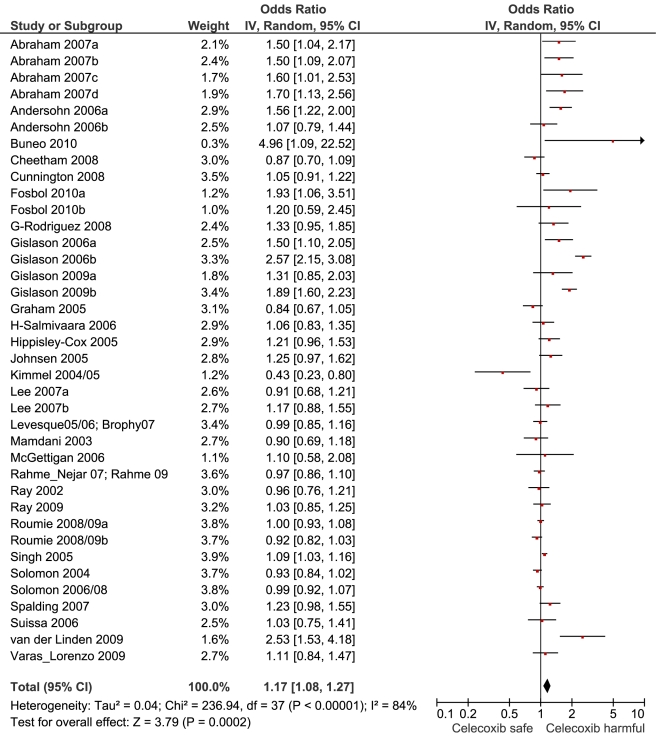
Forest plot for celecoxib.

**Figure 6 pmed-1001098-g006:**
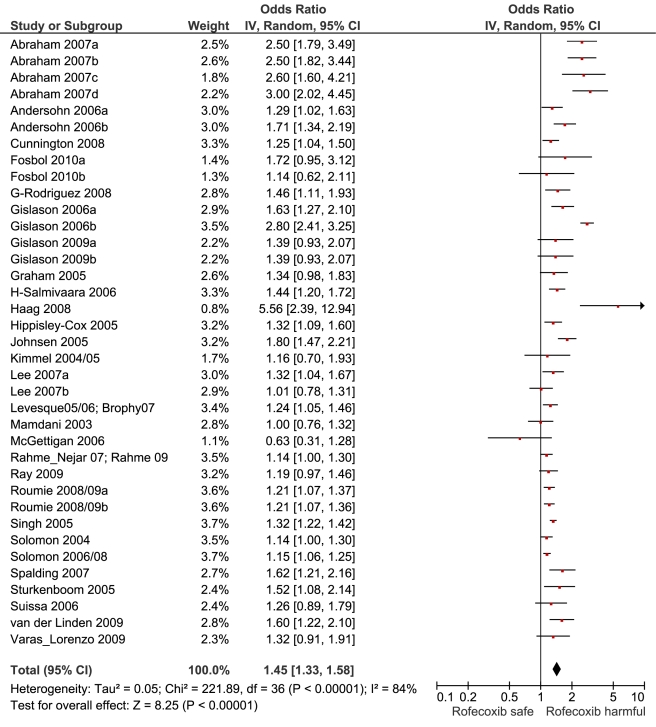
Forest plot for rofecoxib.

**Figure 7 pmed-1001098-g007:**
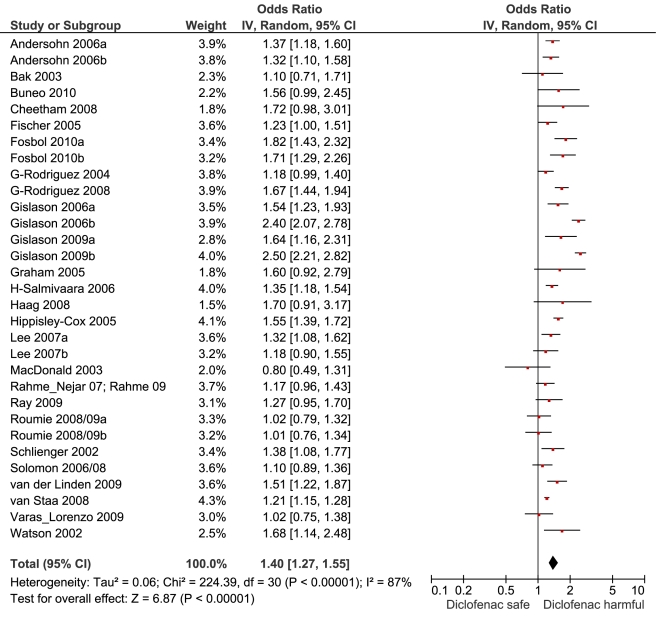
Forest plot for diclofenac.

**Figure 8 pmed-1001098-g008:**
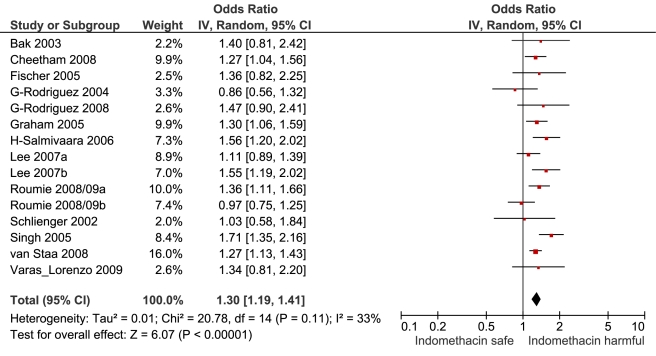
Forest plot for indomethacin.

**Figure 9 pmed-1001098-g009:**
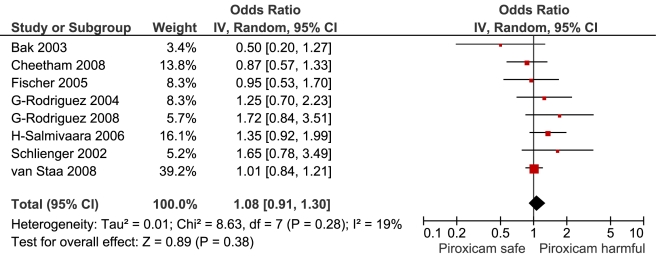
Forest plot for piroxicam.

**Figure 10 pmed-1001098-g010:**
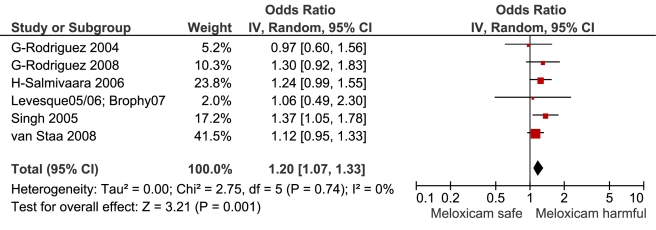
Forest plot for meloxicam.

**Figure 11 pmed-1001098-g011:**
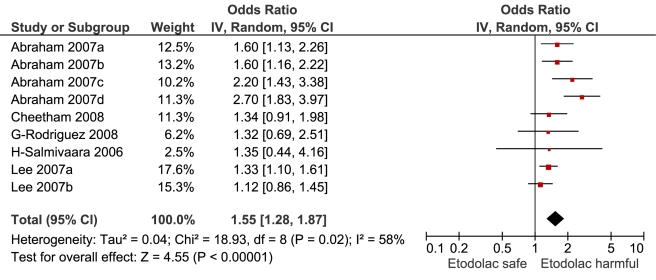
Forest plot for etodolac.

**Figure 12 pmed-1001098-g012:**
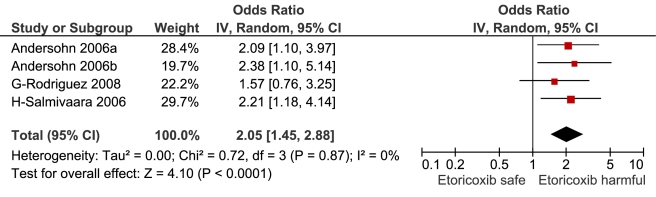
Forest plot for etoricoxib.

**Figure 13:**
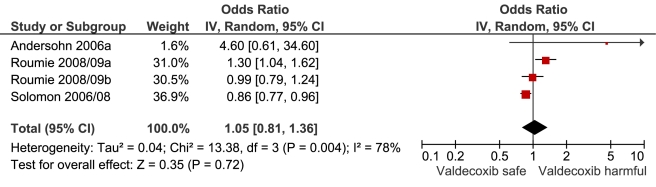
Forest plot for valdecoxib.

**Table 1:** Summary of the numbers of studies and overall results.

Drug	Case-Control Studies		Cohort Studies		Total Number of Studies	Pooled RR (95% CI)	Heterogeneity		
	Number of Studies	Number of Exposed Cases/Controls	Number of Studies	Number of Person-Years of Exposure			Cochran *Q*	*p*-Value	*I* ^2^
Naproxen	24	3,103/24,468	17	159,824	41	1.09 (1.02, 1.16)	143.1	<0.0001	70.70%
Ibuprofen	21	5,716/37,207	17	255,621	38	1.18 (1.11, 1.25)	226.7	<0.0001	81.90%
Celecoxib	20	1,496/12,755	15	179,479	35	1.17 (1.08, 1.27)	236.9	<0.0001	84.40%
Rofecoxib	19	1,662/10,827	15	126,219	34	1.45 (1.33, 1.59)	227.8	<0.0001	84.20%
Diclofenac	16	3,181/13,523	13	50,736	29	1.40 (1.27, 1.55)	224.4	<0.0001	86.60%
Indomethacin	11	788/4,406	3	9,350	14	1.30 (1.19, 1.41)	20.8	0.1	32.60%
Piroxicam	7	288/1,216	1	0^a^	8	1.08 (0.91, 1.30)	8.6	0.3	18.90%
Meloxicam	6	240/714	1	0^a^	7	1.20 (1.07, 1.33)	2.8	0.7	0%
Etodolac	4	464/4,115	1	8,994	5	1.55 (1.28, 1.87)	18.9	0.01	57.70%
Etoricoxib	4	60/116	0	0	4	2.05 (1.45, 2.88)	0.7	0.9	0%
Valdecoxib	1	2/2	4	5,629	5	1.05 (0.81, 1.36)	13.4	0.004	77.60%

a Studies reporting adjusted risk estimates did not all report person-years of exposure.

### Extensively Studied Drugs (Included in Ten or More Studies)

Of the most studied drugs, rofecoxib and diclofenac had the highest overall pooled RR values, and naproxen had the lowest (Figures 3, 6, and 7). Indomethacin was quite close to diclofenac in terms of risk (Table 1; Figures 7 and 8). All analyses of extensively studied drugs (except for indomethacin) were statistically heterogeneous (Table 1). The variable inclusion of studies in the different analyses accounts for the apparent discrepancies between overall RR values given in Table 1 and those given for specific doses, and in “high” and “low” risk populations, in Tables 2 and 3.

**Table 2:** Dose-response relationships for individual drugs included in the analyses.

Information Reported	Rofecoxib		Celecoxib		Ibuprofen		Naproxen		Diclofenac	
	≤25 mg/d	>25 mg/d	≤200 mg/d	>200 mg/d	Low	High	Low	High	Low	High
Overall summary estimates	1.37	2.17	1.26	1.69	1.05	1.78	0.97	1.05	1.22	1.98
95% CI	1.20, 1.57	1.59, 2.97	1.09, 1.47	1.11, 2.57	0.96, 1.15	1.35, 2.34	0.87, 1.08	0.89, 1.24	1.12, 1.33	1.40, 2.82
*p*-Value for dose effect	0.008		0.197		0.0004		0.433		0.009	
Studies contributing dose data	16 of 34 studies reporting on rofecoxib		11 of 35 studies reporting on celecoxib		11 of 38 studies reporting on ibuprofen		10 of 41 studies reporting on naproxen		10 of 29 studies reporting on diclofenac	
Heterogeneity Cochrane *Q*	71.8	80.7	33.7	119.9	43.3	221.4	11.7	29.4	16.3	437.5
*p-*Value	<0.0001	<0.0001	0.0008	<0.0001	<0.0001	<0.0001	0.4	0.0058	0.1786	<0.0001

The RR values in this table differ from those in Table 1 because only a sub-set of all available studies reported dose-response relationships. “Low” and “high” daily doses of ibuprofen, naproxen, and diclofenac were defined in the individual studies as follows. Ibuprofen low dose/high dose: eight studies, ≤1,200 mg/>1,200 mg; one study, ≤1,600 mg/>1,600 mg; two studies, <1,800 mg/≥1,800 mg. Naproxen low dose/high dose: two studies, ≤500 mg/>500 mg; four studies, ≤750 mg/>750 mg; four studies, ≤1,000 mg/≥1,000 mg. Diclofenac low dose/high dose: six studies, ≤100 mg/>100 mg; two, studies <100 mg/≥100 mg; two studies, <150 mg/≥150 mg.

**Table 3:** Estimated RRs of cardiovascular events according to risk of cardiovascular disease.

Information Reported	Drug				
	Rofecoxib	Celecoxib	Ibuprofen	Naproxen	Diclofenac
Low risk population	1.49 (1.28, 1.75)	1.16 (1.02, 1.31)	1.15 (0.99, 1.33)	1.29 (1.09, 1.46)	1.19 (1.07, 1.32)
High risk population	1.54 (1.28, 1.84)	1.17 (1.04, 1.31)	1.32 (1.10, 1.57)	1.23 (1.00, 1.50)	1.14 (0.99, 1.30)
*p-*Value for difference between RR estimates	0.787	0.921	0.242	0.709	0.625
Number of studies contributing data	11	11	6	9	6

Data are given as pooled RR (95% CI). Analyses are from studies that made paired comparisons of cardiovascular risk with individual drugs in low and high risk populations; the definitions of these populations are given in the text, and individual studies are described in Table S1. The RR values in this table differ from those in Table 1 because only a sub-set of all available studies provided data to assess the relationship between RR and background risk of cardiovascular events.

We performed paired analyses of dose effects for five drugs that had been evaluated in ten or more studies (Table 2). Half of the studies reporting on rofecoxib provided information on risk with different doses. For the other drugs, fewer than one-third of studies reported on dose effects. An apparent increase in risk with dose was seen for all drugs except naproxen; this increase in risk was statistically significant for rofecoxib, diclofenac, and ibuprofen, but not for celecoxib. At higher dose levels, there was a doubling or more in risk with rofecoxib and diclofenac. Importantly, of the three drugs available without prescription, ibuprofen and naproxen appeared free of risk at lower doses, in contrast to diclofenac, which was associated with a statistically significant 22% increase in risk at low doses (Table 2). Naproxen was not associated with an increased risk at higher doses, whereas ibuprofen was.

The doses used as cut points are summarised in the footnote to Table 2. In the case of rofecoxib and celecoxib, authors were consistent in reporting doses; increased risks were seen at low doses of both (≤ 25 mg and ≤ 200mg/d, respectively). The majority of studies of ibuprofen defined high doses as more than 1,200 mg/d, above which the drug increased cardiovascular risk by a relative 78%. In the case of diclofenac, the majority of studies used 100 mg/d as the cut point for analysis, above which the drug doubled the risk of cardiovascular events. Though indomethacin was examined in 14 studies, only two reported dose effects, and they used different cut points for analysis, so data pooling was not undertaken.

We categorised studies as including “high risk” or “low risk” individuals based on the risk definitions of the individual studies (Table S1). In general, high risk individuals had experienced prior vascular ischemic events, while low risk individuals had no such history. We were able to obtain paired estimates from low and high risk populations, as outlined in Table 3, which includes the number of studies that contributed data to each comparison. There were no systematic differences in the risk estimates according to background risk of cardiovascular events.

In 12 studies, authors reported events occurring in new users of NSAIDs. Of seven studies reporting on rofecoxib, five found an elevated cardiovascular risk within 30 d of commencement. With celecoxib, risk was evident within 30 d in four of eight studies. In the case of ibuprofen, risk was elevated within 30 d in three of four studies, and with diclofenac in three of four studies. Considering all drugs, nine of 12 studies found cardiovascular risk to be elevated within the first 30 d of use. In three of these studies, the risk was reported to be elevated within a median duration of drug use of 14 d [8],[21],[22].

### Results for Less Extensively Studied Drugs (Included in Fewer than Ten Studies)

This analysis provided an opportunity to examine some less studied drugs. Of these, the highest risk was seen with etoricoxib, investigated in case-control studies only (Table 1; Figure 12). The numbers of cases and controls contributing data were small. Despite this, the lower confidence limit, at 1.45, exceeded that for the other drugs. Valdecoxib was investigated in cohort studies only; a total of 375 events occurred during 12,391 person-years of exposure (Table 1; Figure 13). This drug was not associated with increased risk of cardiovascular events. Etodolac was studied more extensively and in the unpaired analyses appeared to have a profile similar to that of rofecoxib (Table 1; Figure 2). Meloxicam and piroxicam were not widely investigated (Figures 9 and 10). In the pooled analyses, meloxicam had a risk profile similar to that of ibuprofen and celecoxib, while piroxicam appeared similar to naproxen.

### Pair-Wise Comparisons

The results of the pair-wise comparisons are shown in Table 4. Etoricoxib had a significantly higher RR than either ibuprofen or naproxen; the point estimates for the RRs suggest it also had a higher risk than either rofecoxib or diclofenac, but this was not statistically significant at a *p*-value of 0.01. More data were available for etodolac. Despite its similarity to rofecoxib in the unpaired comparisons, it was indistinguishable from diclofenac, naproxen, and ibuprofen in pair-wise comparisons.

**Table 4:** Selected pair-wise comparisons of individual drugs.

Drug Tested	Reference Drug in the Comparison				
	Rofecoxib	Diclofenac	Ibuprofen	Naproxen	Celecoxib
**Etoricoxib**	1.29 (0.86, 1.93), *n = *3 studies	1.36 (0.89, 2.09), *n = *3 studies	**1.68** (1.14, 2.49), *n = *3 studies	**1.75** (1.16, 2.64), *n = *3 studies	
**Etodolac**		0.95 (0.78, 1.16), *n = *5 studies	1.04 (0.88, 1.24), *n = *7 studies	1.10 (0.96, 1.26), *n = *7 studies	
**Diclofenac**	1.0 (0.89, 1.12), *n = *18 studies		**1.13** (1.03, 1.24), *n = *27 studies	**1.22** (1.11, 1.35), *n = *25 studies	**1.15** (1.02, 1.30), *n = *19 studies
**Naproxen**			**0.92** (0.87, 0.99), *n = *32 studies	__	0.96 (0.81, 1.13), *n = *23 studies
**Meloxicam**				**1.11** (1.0, 1.23), *n = *6 studies	
**Indomethacin**				**1.23** (1.10, 1.39), *n = *15 studies	

Values are pooled RRRs and 99% CIs. Bold indicates significant difference at *p*<0.0033 (the Bonferroni-adjusted threshold *p-*value; *n* = 15 comparisons; alpha = 0.05).

Because of growing concerns about risk with diclofenac, we performed several comparisons. It had a risk identical to that of rofecoxib and had a significantly higher RR than celecoxib, naproxen, or ibuprofen.

We thought it was clinically relevant to determine whether naproxen had any advantage over two other allegedly low risk drugs, celecoxib and ibuprofen. The summary data show a small but statistically significant advantage of naproxen over ibuprofen. In contrast, the overall risks with naproxen and celecoxib appear very similar.

We also performed pair-wise comparisons of less well studied drugs. The RR with meloxicam was about 10% higher than with naproxen. The *p*-value for this comparison (0.012) did not reach the Bonferroni-adjusted threshold (Table 4). In contrast, indomethacin had a statistically significant 23% increase in RR compared with naproxen.

### Sensitivity Analyses

The results of the sensitivity analyses for the pair-wise comparisons are given in Table 5. With the exception of the comparison between diclofenac and ibuprofen, a hypothetical unmeasured confounding variable would need to have an association with the outcome with a RR of 2.0 or greater (or its reciprocal, 0.5) in order to bias a true null result to the observed.

**Table 5:** Results of sensitivity analyses on selected pair-wise comparisons.

Comparison	RRR	RR_CD_	P_C1_	P_C0_	RRR_adj_	Percent Bias
Etoricoxib versus naproxen	1.75	11.00	0.25	0.10	1.00	75.00
Etoricoxib versus ibuprofen	1.68	9.40	0.25	0.10	1.00	68.48
Indomethacin versus naproxen	1.23	2.80	0.25	0.10	1.00	22.88
Diclofenac versus naproxen	1.22	2.70	0.25	0.10	1.00	21.79
Diclofenac versus celecoxib	1.15	2.10	0.25	0.10	1.00	14.86
Diclofenac versus ibuprofen	1.13	1.95	0.25	0.10	1.00	13.01
Naproxen versus ibuprofen	0.92	0.50	0.25	0.10	1.00	−7.89

RR_CD_ is the association between confounder and disease outcome. P_C1_is the prevalence of confounder in the exposed. P_C0_ is the prevalence of confounder in the unexposed. RRR_adj_ is the “true”, or fully adjusted, RRR. Percent bias is the percentage change to the RRR that would be introduced by a hypothetical confounding variable under the assumptions in the table.

## Discussion

This updated systematic review of pharmaco-epidemiological studies of the cardiovascular risks of NSAIDs correlates broadly with several meta-analyses of randomised controlled trials [9],[10]. It contributes new information on some familiar drugs, and provides potentially important information on some little studied agents.

### Commonly Studied Drugs

The highest overall risks were seen with rofecoxib and diclofenac and the lowest with ibuprofen and naproxen. Naproxen was risk-neutral at all doses and had a significantly lower RR than ibuprofen in a pair-wise comparison. Evaluation of dose effects with rofecoxib, celecoxib, diclofenac, ibuprofen, and naproxen, and the results of the pair-wise analyses, enabled a more comprehensive assessment of the comparative risk of these popular medications. The last three are available without prescription, and the implications of this are discussed below. In the dose analyses, the similarity between rofecoxib and diclofenac persisted, and in neither case did the data define a “safe” lower dose. Celecoxib had an elevated risk overall and at both low (≤200 mg/d) and high doses (>200 mg/d); data for doses in excess of 200 mg/d were sparse in these pharmaco-epidemiological studies. However, meta-analyses of the results of randomised controlled trials have shown a clear and substantial increase in risk at daily doses of 400 mg or more [23],[24].

The data here suggest that naproxen is superior to ibuprofen in terms of cardiovascular safety. The apparent safety of naproxen is well reported, but to our knowledge this is the first evidence to show a significant difference between these two drugs. A recently published network meta-analysis of randomised trials also found naproxen to be the safest choice, and found higher levels of risk with ibuprofen, particularly for stroke [10]. However, just two randomised trials contributed data for ibuprofen, high doses were used (2,400 mg/d), and there were only 45 cardiovascular events among users [10]. As we showed here, dose is critical with ibuprofen but apparently not with naproxen.

In the pair-wise analyses, celecoxib was indistinguishable from naproxen. Nevertheless, in the overall analyses, and in the investigations of dose, it was associated with statistically significant risk increases. The advantage of naproxen over ibuprofen and celecoxib is small and must be balanced with its gastrointestinal risks [6]. However, in our view, it remains the safest choice when NSAIDs need to be used in patients at high risk of cardiovascular events. In making this statement we are taking account of the overall pooled RR value, the lack of a dose-response relationship, naproxen's superiority in the pair-wise comparisons, and the consistency of these findings with its pharmacology—having high inhibitory activity for Cox-1.

### Less Commonly Studied Drugs

Data were sparse for etoricoxib. It is similar in its chemical structure and pharmacology to rofecoxib; it had a higher RR for cardiovascular events than ibuprofen and naproxen in pair-wise comparisons, and had a non-significantly greater RR value than diclofenac and rofecoxib. This is consistent with the findings of the MEDAL randomised trial program, which found similar levels of cardiovascular risk with etoricoxib and diclofenac [25]. A network meta-analysis of randomised trials also found etoricoxib to have the highest RR of cardiovascular death, but did not find an increased risk of myocardial infarction, stroke, or a composite cardiovascular outcome [10]. Our analyses were based on small numbers of events (Table 1), and we are unable to resolve this apparent contradiction.

Valdecoxib, like etoricoxib, is a highly Cox-2-selective agent, which was withdrawn from the market in 2005 after excessive numbers of cardiovascular events were reported among post-operative cardiac surgery patients [26]. In this meta-analysis, it was not associated with increased overall risk. The contributing studies were undertaken among two populations that included Medicare and Medicaid patients in the United States (see references A37, A38, A45, and A46 in Text S2). While both populations had underlying cardiovascular risks, a review of event rates among different sub-groups showed valdecoxib was comparable with other agents (see reference A38 in Text S2). We are not able to resolve the apparent discrepancy between randomised and non-randomised data, which may be due to the play of chance.

This review provides information on four drugs that were not included in previous meta-analyses of randomised trial data [9]–[11] and have been little studied in relation to their cardiovascular safety: piroxicam, etodolac, indomethacin, and meloxicam. Piroxicam is similar to naproxen in being a selective inhibitor of Cox-1 [27]. In these analyses it had a cardiovascular risk profile similar to that of naproxen. However, its use in recent years has declined because of concerns about the very high risk of serious gastrointestinal events [6]. It is hard to believe that the data reviewed here will reverse its declining market share.

Etodolac was studied more extensively and in the unpaired analyses had a risk profile similar to that of rofecoxib. However, the pair-wise analyses are likely to be less confounded, and these analyses showed etodolac to be similar to two low risk drugs, ibuprofen and naproxen.

Indomethacin appears to have a risk profile similar to that of diclofenac in both unpaired and pair-wise analyses and is associated with a high risk of gastrointestinal damage, as well as adverse effects in the central nervous system [19],[28],[29]. It is still commonly recommended in the treatment of gout, but there appear to be no good reasons to retain it in clinical practice.

The data on meloxicam are sparse; although it had a higher RR than naproxen in the pair-wise analysis, this difference was not statistically significant. However, the *p-*value for this comparison, although not meeting the Bonferroni-adjusted threshold, was close to 0.01. In other respects, meloxicam appears similar to ibuprofen and celecoxib, and probably has a level of risk of serious gastrointestinal events similar to that of diclofenac [30]. However, taking account of its relative Cox-2 selectivity and the data presented here, we believe that it should be avoided in patients at high risk of cardiovascular events.

### Importance of Background Risk

The balance of the evidence from the pharmaco-epidemiological data summarised here suggests that background risk of cardiovascular events does not modify the RR in users of these drugs. In other words “low risk” individuals are exposed to the same proportional increase in the probability of an adverse event as those at high background risk, but their excess risk will be lower. The significance of this finding in relation to potential channelling effects on cardiovascular risk is discussed below. These findings are at odds with the results of an individual patient meta-analysis of randomised placebo-controlled trials of celecoxib [23], which found that both absolute risk and RR increased with background risk of cardiovascular disease. This was based on an analysis of high doses of celecoxib, and there were only 52 events in the exposed groups.

### Timing of Increase in Risk on Treatment

Duration of use was difficult to study because administrative datasets include information on prescribing or dispensing, not consumption, of drugs. Accordingly, few studies quantified risk with duration of use of less than one month. This review confirmed increases in risk within the first month of treatment for some of the drugs reviewed here. This conclusion is supported by an evaluation of the duration of NSAID treatment and associated cardiovascular risk among a Danish cohort of patients with prior myocardial infarction [31], which was published after the completion of our literature review. Treatment with NSAIDs was associated with early risk of recurrent infarction or death; risk with rofecoxib was increased after 7–14 d of treatment, with celecoxib after 14–30 d, and with ibuprofen after 7 d. Diclofenac increased risk from the beginning of treatment and had the highest risk, with a hazard ratio of 3.26 (95% CI 2.57, 3.86). Early onset of risk with NSAIDs is further supported by randomised studies that have reported increases in risk after brief exposure, and by the short time course of the biological mechanisms thought to be the cause of this adverse event [32],[33].

### Estimating the Effects of Non-Prescription Use of NSAIDs

In considering the risk associated with non-prescription use of NSAIDs, it is important to consider three factors: safety at low doses, with short durations of treatment, and in populations with a low background risk of cardiovascular events. The maximum recommended daily doses for non-prescription use in the United Kingdom, as an example, are as follows: ibuprofen, 1,200 mg/d; naproxen, 750 mg/d; and diclofenac, 75 mg/d. Our risk estimates are based on prescription data, not a survey of non-prescription drug users, and the variable dose cut points used by authors made interpretation of dose effects difficult. But 1,200 mg/d or less of ibuprofen appears to be free of risk in the data shown here, and the cardiovascular risk with naproxen was not significantly elevated with high or low doses. In contrast, the lower prescription doses of diclofenac, which were associated with increased risk, fall close to the maximum daily recommended dose for non-prescription use. Eight of the ten studies that included analyses of low doses of diclofenac defined these as 100 mg/d or less, a range that is close to the maximum recommended dose for non-prescription diclofenac products (75 mg). The higher doses of ibuprofen (with higher risk) may be reached relatively easily, particularly with the 400-mg strength preparations that are available without prescription in some countries.

### Limitations of the Work

There are a number of limitations to this work. We relied on observational studies, which are subject to a range of biases. Quantitatively, many of the differences in RR between individual drugs were small and in a range that might be explained by residual confounding. However, sensitivity analyses showed that the majority of the significant pair-wise comparisons were fairly robust. The exception was the comparison of diclofenac and ibuprofen. We did not have access to the individual patient data and were therefore limited to the adjustment procedures used by the investigators. However, we believe that a high degree of confounding by indication is unlikely with these drugs, as they are used in very similar circumstances, there are clear dose effects for most drugs, and we adjusted for confounding at study level by performing some key pair-wise within-study analyses. Also, the ranking of risks correlates fairly closely with what has been seen in meta-analyses of randomised trials [9],[10],[23],[24]. It is known that there is a degree of channelling of non-steroidal anti-inflammatory agents, meaning drugs that have a lower risk of gastrointestinal adverse effects, such as rofecoxib and celecoxib, may have been prescribed preferentially to those patients perceived to be at high risk of this complication [34]. There is a correlation between risk factors for gastrointestinal and cardiovascular complications. Although these factors will increase the background risk of cardiovascular events, and the excess risk attributable to the drugs, as shown here, they do not appear to influence the RRs.

Most of the studies included here relied on definitions of exposure and outcomes derived from large linked administrative databases or electronic health records. Neither source type is designed for research, and key information, particularly use of non-prescription NSAIDs and aspirin, is usually not recorded. In addition, information on cardiovascular risk factors is limited, as smoking history is often not recorded, and the results of laboratory tests are generally not included in the databases. The adjustments made in these studies relied on other measures, such as a history of cardiovascular events and prescriptions written for diabetes, hypertension, and hyperlipidaemia.

Heterogeneity was very significant in many of the analyses we conducted. We looked extensively for causes of heterogeneity, by studying variation in RR with dose, background risk, and year of publication (data not shown). Within these sub-group analyses, heterogeneity was common. The most important association with heterogeneity was the amount of available statistical information—heterogeneity was more significant with extensively studied drugs (Table 1). This may be due to the fact that the individual RR estimates for these commonly used drugs were very precise, as they were estimated in large population databases. Consequently, heterogeneity statistics tended to be significant even though the differences between the study-specific estimates were small.

While high quality randomised studies provide the least biased estimates of treatment effects, they are not commonly used to investigate adverse effects. Meta-analyses of randomised data on adverse events, preferable in theory to observational data, may be constrained by small numbers of events [9]–[11],[23],[24]. Some reassurance of the accuracy of observational data is provided by a recent comparison of estimates of harm from meta-analyses of randomised studies with those from meta-analyses of observational studies, which found risk estimates for the events investigated to be concordant [35]. That analysis included data from the earlier version of this review.

### Conclusions

Despite the limitations of the data, the large sizes of the studies reviewed here, the presence of consistent dose-response relationships, and general agreement with the results of randomised trials give us confidence in the results. In our view, the results are sufficiently robust to inform clinical and regulatory decisions. From a clinical perspective, naproxen and low-dose ibuprofen have the most favourable cardiovascular risk profiles. This advantage has to be weighed against the drugs' gastrointestinal risks, and for ibuprofen, avoidance of antagonism of aspirin's beneficial effect [36]. While celecoxib was indistinguishable from naproxen in pair-wise comparisons, the more extensive dose data available in the randomised trials, and a consideration of its relative Cox-2 selectivity, makes us reluctant to recommend it in patients at risk of cardiovascular events. The data for etoricoxib are limited but raise serious concerns about its safety, particularly as analogues such as rofecoxib and lumiracoxib have already been withdrawn. The review supports the calls for regulatory action on diclofenac, particularly as it is available without prescription in several countries. The data here show an elevation of risk with low doses, unlike its competitor drugs. In the case of ibuprofen, labelling warnings should be strengthened to stop patients at high background risk of cardiovascular disease exceeding the maximum recommended dose for non-prescription use of 1,200 mg/d. The review also casts doubts on the safety of an older drug, indomethacin. Indomethacin has a range of gastrointestinal and central nervous system effects that, combined with the evidence presented here on cardiovascular risk, should lead to questioning of its continued clinical use.

## Supporting Information

Table S1 Details of all studies included in the meta-analysis.
(DOC)

Text S1 Summary of terms used in the literature search.
(DOC)

Text S2 References for studies included in the systematic review and studies excluded from the review.
(DOC)

## Abbreviations

CI, confidence interval; NSAID: non-steroidal anti-inflammatory drug
RR: relative risk
RRR: ratio of relative risks
