# Supplementary material for: Cardiovascular Risk with Non-Steroidal Anti-Inflammatory Drugs: Systematic Review of Population-Based Controlled Observational Studies
Source: PLoS Med. 2011 Sep 27;8(9):e1001098. doi: 10.1371/journal.pmed.1001098 (PMC3181230; doi:10.1371/journal.pmed.1001098)
Supplement: Table S1 — Details of all studies included in the meta-analysis. (DOC) [file pmed.1001098.s001.doc]

**Table S1 Details of all studies included in the meta-analysis**

1. **Case-control studies**

BMI = body mass index; CHD = coronary heart disease; HRT = hormone replacement therapy; OTC = over the counter.

*Levesque 2006 & Brophy 2007 used same dataset as Levesque 2005, included in our 2006 meta-analysis, but provide new information on risk.

# Provided risk estimates for individuals at 'Low Risk' and 'High Risk' for cardiovascular events.

** Provided risk estimates for cardiovascular events among new users of NSAIDs.

Outcomes: Fatal, Non-fatal, Death are specified for studies where we could be certain, or reasonably so, that these outcomes were assessed.

Reference category: No use / remote use of NSAIDs.

**B) Cohort studies**

BMI = body mass index; CHD = coronary heart disease; OTC = over the counter.

#Provided risk estimates for individuals at 'Low Risk' and 'High Risk' for cardiovascular events.

** Provided risk estimates for cardiovascular events among new users of NSAIDs.

Outcomes: Fatal, Non-fatal, Death are specified for studies where we could be certain, or reasonably so, that these outcomes were assessed.

Reference category = NSAID/coxib non-use in all studies except Refs 40 & 41 (Rahme et al) where reference was acetaminophen use.
