# Supplementary material for: Cardiovascular Risk with Non-Steroidal Anti-Inflammatory Drugs: Systematic Review of Population-Based Controlled Observational Studies
Source: PLoS Med. 2011 Sep 27;8(9):e1001098. doi: 10.1371/journal.pmed.1001098 (PMC3181230; doi:10.1371/journal.pmed.1001098)
Supplement: Text S1 — Summary of terms used in the literature search. (DOC) [file pmed.1001098.s002.doc]

**Text S1 Summary of terms used in the literature search**

Non-Steroidal Anti-Inflammatory Drugs

NSAIDs

Selective Non-Steroidal Anti-Inflammatory Drugs

Non-Selective Non-Steroidal Anti-Inflammatory Drugs

Cyclo-oxygenase-2 inhibitors

COX-2 inhibitors

Selective Cyclo-oxygenase-2 inhibitors

Selective COX-2 inhibitors

Acute coronary syndrome

Myocardial infarction

Vascular ischemia

Cardiovascular ischemia

Cardiovascular risk

Cardiovascular disease

Cardiovascular side effects

Coronary heart disease

Cerebrovascular disease

Cerebrovascular ischemia

Cerebrovascular accident

Stroke

Observational study

Case Control study

Cohort study

Controlled study

Epidemiological study

Celecoxib

Rofecoxib

Meloxicam

Lumiracoxib

Valdecoxib

Parecoxib

Etoricoixib

Diclofenac

Ibuprofen

Naproxen

Piroxicam

Indomethacin

Indometacin

Azapropazone

Etodolac

Fenbufen

Fenoprofen

Flurbiprofen

Ketoprofen

Ketorolac

Mefenamic Acid

Nabumetone

Sulindac

Tenoxicam

Tiaprofenic Acid
