# Supplementary material for: Cardiovascular Risk with Non-Steroidal Anti-Inflammatory Drugs: Systematic Review of Population-Based Controlled Observational Studies
Source: PLoS Med. 2011 Sep 27;8(9):e1001098. doi: 10.1371/journal.pmed.1001098 (PMC3181230; doi:10.1371/journal.pmed.1001098)
Supplement: Text S2 — References for studies included in the systematic review and studies excluded from the review. (DOC) [file pmed.1001098.s003.doc]

**Text S2**

**References for studies included in the systematic review and studies excluded from the review**

**Included Studies**

**Case control studies**

1. Garcia Rodriguez LA, Varas-Lorenzo C, Patrono C. Differential effects of aspirin and non-aspirin non-steroidal anti-inflammatory drugs in the primary prevention of myocardial infarction in post-menopausal women. Epidemiology 2000; 11:382-7.
2. Schlienger RG, Jick H, Meier CR. Use of non-steroidal anti-inflammatory drugs and the risk of first-time acute myocardial infarction. Br J Clin Pharmac 2002; 54:327-332.
3. Solomon DH, Glynn RJ, Levin R, Avorn J. Non-steroidal anti-inflammatory drug use and acute myocardial infarction. Arch Intern Med 2002; 162:1099-104.
4. Watson DJ, Rhodes T, Cai B, Guess HA. Lower risk of thromboembolic cardiovascular events with naproxen among patients with rheumatoid arthritis. Arch Intern Med 2002; 162:1105-110.
5. Bak S, Andersen M, Tsiropoulos I, Garcia Rodriguez LA, Hallsa J, Christensen K, Gaist D. Risk of stroke associated with non-steroidal anti-inflammatory drugs. Stroke 2003; 34:379-86.
6. Garcia Rodriguez LA, Varas-Lorenzo C, Maguire A, Gonzales-Perez A. Non-steroidal anti-inflammatory drugs and the risk of myocardial infarction in the general population. Circulation 2004; 109:3000-6.
7. Garcia Rodriguez L, Gonzalez-Perez A. Long-term use of non-steroidal anti-inflammatory drugs and the risk of myocardial infarction in the general population. BMC Medicine 2005, 3:17 doi:10.1186/1741-7015-3-17.
8. Solomon DH , Schneeweiss S, Glynn RJ, Kiyota Y, Levin R, Mogun H, Avorn J Relationship between selective cyclooxygenase-2 inhibitors and acute myocardial infarction in older adults. Circulation 2004; 109:2068-73.
9. Fischer LM, Schlienger RG, Matter CM, Jick H, Meier CR. Current use of non-steroidal anti-inflammatory drugs and risk of acute myocardial infarction. Pharmacotherapy 2005; 25:503-10.
10. Graham DJ, Campen D, Hui R, Spence M, Cheetham C, Levy G, Shoor S, Ray WA. Risk of acute myocardial infarction and sudden cardiac death in patients treated with cyclooxygenase-2 selective and non-selective non-steroidal anti-inflammatory drugs: nested case-control study. Lancet. 2005; 365(9458): 475-81.
11. Hippisley-Cox J, Coupland C. Risk of myocardial infarction in patients taking cyclooxygenase inhibitors or conventional non-steroidal anti-inflammatory drugs: population based nested case-control analysis. BMJ 2005; 330:1366-72.
12. Johnsen SP, Larsson H, Tarone RE, McLaughlin JK, Nørgård B, Friis S, Sørensen HT. Risk of hospitalisation for myocardial infarction among users of rofecoxib, celecoxib and other NSAIDs: A population based case control study. Arch Intern Med 2005; 165:978-84.
13. Kimmel SE, Berlin JA, Reilly M, Jaskowiak J, Kishel L, Chittams J, Strom BL. The effects of non-selective non-aspirin non-steroidal anti-inflammatory medications on the risk of non-fatal myocardial infarction and their interaction with aspirin. J Am Coll Cardiol 2004; 43:985-90.
14. Kimmel SE, Berlin JA, Reilly M, Jaskowiak J, Kishel L, Chittams J, Strom BL Patients exposed to rofecoxib and celecoxib have different odds of nonfatal myocardial infarction. Ann Int Med 2005; 142(7):157-64.
15. Levesque LE, Brophy JM, Zhang B. The risk for myocardial infarction with cyclooxygenase-2 inhibitors: a population study of elderly adults. Ann Int Med 2005; 142(7):481-9.
16. Singh G, Mithal A, Triadafilopoulos G. Both selective COX-2 inhibitors and non-selective NSAIDs increase the risk of acute myocardial infarction in patients with arthritis: selectivity is with the patient not the drug class. Ann Rheum Dis 2005; 64(Suppl III)85.
17. Sturkenboom MCJM, Dieleman J, Verhamme K, Straus S, Vander Hoeven-Borgman M, van der Lei J. Cardiovascular events during use of COX-2 selective and non-selective NSAIDs. Pharmacoepidemiology & Drug Safety 2005; 14:S57.
18. McGettigan P, Han P, Henry D. Cyclooxygenase-2 inhibitors and coronary occlusion: exploring dose-response relationships. Br J Clin Pharmacol 2006. DOI:10.1111/j.1365-2125.2006.02660.x.
19. Andersohn F, Suissa S, Garbe E. Use of first-and second-generation cyclooxygenase-2-selective non-steroidal anti-inflammatory drugs and risk of acute myocardial infarction. Circulation 2006; 113:1950-57.
20. Andersohn F, Schade R, Suissa S, Garbe E. Cyclooxygenase-2 selective non-steroidal anti-inflammatory drugs and the risk of ischemic stroke: a nested case-control study. Stroke 2006; 37:1725-30.
21. Helin-Salmivaara A, Virtanen A, Vesalainen R, Gronroos JM, Klaukka T, Idanpaan-Heikkila J, Huupponen R. NSAID use and the risk of hospitalization for first myocardial infarction in the general population: a nationwide case-control study from Finland. Eur Heart J 2006; 26:1657-63.
22. Suissa S, Bernatsky S, Hudson M. Antirheumatic drug use and the risk of acute myocardial infarction. Arth Rheum 2006; 55:531-36.
23. Levesque L, Brophy J, Zhang B. Time variations in the risk of myocardial infarction among elderly users of Cox-2 inhibitors. CMAJ 2006; 174(11). DOI:10.1503/cmaj.051679.
24. Brophy JM, Levesque LE, Zhang B. The coronary risk of cyclo-oxygenase-2 inhibitors in patients with a previous myocardial infarction. Heart 2007; 93:189-94.
25. Lee TA, Bartle B, Weiss KB. Impact of NSAIDS on mortality and the effect of preexisting coronary artery disease in US veterans. Am J Med 2007; 120:98e9-e16.
26. Cheetham T, Graham D, Campen D, Hui R, Spence M, Levy G, Shoor S. Myocardial Infarction and Its Association with the Use of Nonselective NSAIDs: A Nested Case-Control and Time-to-Event Analysis. The Permanente Journal 2008; 12(1):16-22.
27. Garcia Rodriguez L, Tacconelli S, Patrignani P. Role of dose potency in the prediction of myocardial infarction associated with non-steroidal anti-inflammatory drugs in the general population. J Am Coll Cardiol 2008; 52:1628-36.
28. van der Linden MW, van der Bij S, Welsing P, Kuipers EJ, Herings R. The balance between severe cardiovascular and gastrointestinal events among users of selective and non-selective non-steroidal anti-inflammatory drugs. Ann Rheum Dis 2009 68: 668-673.
29. Varas-Lorenzo C, Castellsague J, Stang MR, Perez-Guthann S, Aguado J, Garcia Rodriguez L. The use of selective cyclooxygenase-2 inhibitors and the risk of acute myocardial infarction in Saskatchewan, Canada. P’Epi Drug Saf 2009; 18:1016-25.
30. Buneo H, Bardaji A, Patrignani P, Martin-Merino E, Garcia Rodriguez L. Use of non-steroidal anti-inflammatory drugs and type-specific risk of acute coronary syndrome. Am J Cardiol 2010; 105:1102-06.

**Cohort studies**

1. Ray WA, Stein CM, Hall K, Daugherty JR, Griffin MR. Non-steroidal anti-inflammatory drugs and the risk of serious coronary heart disease: an observational cohort study. Lancet 2002; 359:118-23.
2. Ray WA, Stein CM, Daugherty JR, Hall K, Arbogast PG, Griffin MR. Cyclo-oxygenase-2 selective non-steroidal anti-inflammatory drugs and risk of serious coronary heart disease. Lancet 2002; 360:1071-3.
3. Curtis JP, Wang Y, Portnay E, Masoudi F, Havranek E, Krumholz H. Aspirin, ibuprofen, and mortality after myocardial infarction: retrospective cohort study. BMJ 2003; 327:1322-3.
4. MacDonald TM, Wei L. Effect of ibuprofen on cardio-protective effect of aspirin. Lancet 2003; 361:573-4.
5. Mamdani M, Rochon P, Juurlink DN, Anderson GM, Kopp A, Naglie G, Austin PC, Laupacis A. Effect of selective cyclooxygenase-2 inhibitors and naproxen on short-term risk of acute myocardial infarction in the elderly. Arch Int Med 2003; 163(4):481-6.
6. Gíslason GH, Jacobsen S, Buch P, Rasmussen JN, Friberg J, Abildstrom SZ, Torp-Pedersen C. Increased mortality related to treatment with selective Cyclo-oxygenase-2 inhibitors and Non-steroidal anti-inflammatory drugs after acute myocardial infarction**.** Circulation 2006; 113:2906-13.
7. Solomon DH, Avorn J, Sturmer T, Glynn R, Mogun H, Schneeweiss S. Cardiovascular outcomes in new users of coxibs and NSAIDs: Subgroup analyses to determine cardiovascular risk associated with non-steroidal anti-inflammatory drugs and coxibs in specific patient groups. Arth Rheum 2006; 54:1378-89.
8. Solomon DH, Glynn R, Rothman K, Schneeweiss S. Setoguchi S, Mogun H, Avorn J, Sturmer T. Sub-group analyses to determine cardiovascular risk associated with non-steroidal antiinflammatory drugs and coxibs in specific patient groups. Arth Rheum 2008; 59:1097-1104.
9. Abraham NS, El-Serag HB, Hartman C, Richardson P, Deswal A. Cyclooxygenase-2 selectivity of non-steroidal anti-inflammatory drugs and the risk of myocardial infarction and cerebrovascular accident. Aliment Pharmacol Ther 2007; 25:913-24.
10. Rahme E, Nedjar H. Risks and benefits of COX-2 inhibitors *vs* non-selective NSAIDS: does their cardiovascular risk exceed their gastrointestinal benefit? A retrospective cohort study. Rheumatology 2007; 46:435-438.
11. Rahme E, Watson D, Kong S, Toubouti Y, LeLorier J. Association between non-naproxen NSAIDs, Cox-2 inhibitors and hospitalisation for acute myocardial infarction among the elderly: a retrospective cohort study. P’Epi Drug Saf 2007; 16:493-503.
12. Spalding WM, Reeves MJ, Whelton A. Thromboembolic cardiovascular risk among arthritis patients using cyclooxygenase-2-selective inhibitor or non-selective cyclooxygenase inhibitor non-steroidal anti-inflammatory drugs. Am J Ther 2007; 14:3-12.
13. Cunnington M, Webb D, Qizilbash N, Blum D, Mander A, Funk MJ, Weil J. Risk of ischaemic cardiovascular events from selective cyclooxygenase-2 inhibitors in osteoarthritis. P’Epi Drug Saf 2008; 17:601-8.
14. Haag M, Bos M, Hofman A, Koudstaal P, Breteler M, Stricker B. Cysoooxygenase selectivity of non-steroidal anti-inflammatory drugs and risk of stroke. Arch Int Med 2008; 168:1219-24.
15. Roumie C, Mitchel E, Kaltnebach L, Arbogast P, Gideon P, Griffin M. Non-aspirin NSAIDs, cyclooxygenase-2 inhibitors, and the risk for stroke. Stroke 2008; 39:2037-45.
16. Roumie C, Choma N, Kaltnebach L, Mitchel E, Arbogast P, Griffin M. Non-aspirin NSAIDs, cyclooxygenase-2 inhibitors and risk for cardiovascular events- stroke, acute myocardial infarction, and death from coronary heart disease. P’Epi Drug Saf 2009; 18:1053-63.
17. Van Staa TP, Reitbrock S, Setakis E, Leufkens H. Does the varied use of NSAIDs explain the differences in the risk of myocardial infarction? J Int Med 2008; 264:481-92.
18. Fosbol EL, Gislason GH, Jacobsen S, Folke F, Hansen ML, Schramm TK, Sorensen R, Rasmussen JN, Andersen S, Abildstrom S, Traerup J, Poulsen H, Kober L, Torp-Pedersen C, Rasmussen S. Risk of myocardial infarction and death associated with the use of non-steroidal anti-inflammatory drugs (NSAIDs) among healthy individuals: a nationwide cohort study. Clin Pharmacol Ther 2009; 85:190-97.
19. Fosbol EL, Folke F, Gislason GH, Jacobsen S, Rasamussen JN, Sorensen R, Schramm TK, Andersen S, Rasmussen S, Poulsen H, Kober L, Torp-Pedersen C, Gislason G. Cause-specific cardiovascular risk associated with non-steroidal anti-inflammatory drugs among healthy individuals. Circ Cardiovasc Qual Outcomes 2010; 3: DOI: 10.1161/circoutcomes.109.861104.
20. Gislason G, Rasmussen JN, Abildstrom SZ, Schramm TK, Hansen ML, Fosbol EL, Sorensen R, Folke F, Buch P, Gadsboll N, Rasmussen S, Poulsen H, Kober L, Madsen M, Torp-Pedersen C. Increased mortality and cardiovascular morbidity associated with use of nonsteroidal anti-inflammatory drugs in chronic heart failure. Arch Int Med 2009; 169:141-9.
21. Ray W, Varas-Lorenzo C, Chung C, Castellsague J, Murray K, Stein M, Daugherty J, Arbogast P, Garcia Rodriguez L. Cardiovascular risks of non-steroidal anti-inflammatory drugs in patients after hospitalization for serious coronary heart disease. Circ Cardiovasc Qual Outcomes 2009; 2:155-63.

**Excluded Studies: categorized by reasons for exclusion**

**No non-use/remote use comparator group**

1. Huang W, Hsiao F, Wen Y, Tsai Y. Cardiovascular events associated with the use of four non-selective NSAIDS (etodolac, nabumetone, ibuprofen or naproxen) versus a cyclooxygenase-2 inhibitor (celecoxib): A population-based analysis in Taiwanese adults. Clin Ther 2006; 28:1827-36.
2. Huang W, Hsiao F, Tsai Y, Wen Y, Shis Y. Cardiovascular events associated with long term use of celecoxib, rofecoxib and meloxicam in Taiwan. Drug Saf 2006; 29(3):261-272.
3. Laharie D, Droz-Perroteau C, Benichou J, Amouretti M, Blin P, begaud B, Guiard E, Dutoit S, Lamarque S, Moride Y, Depont F, Fourrier-Reglat A, Moore N. Hospitalisations for gastrointestinal and cardiovascular events in the CADEUS cohort of traditional or coxib NSAID users. Br J Clin Pharmac 2010; 69(3):295-302.
4. Motsko SP, Rascatti KL, Busti AJ, Wilson JP, Barner JP, Lawson KA, Worchel J. Temporal relationship between use of NSAIDs, including selective COX-2 inhibitors, and cardiovascular risk. Drug Saf 2006; 29(7):621-632.
5. Warner JJ, Weideman RA, Kelly KC, Brilakis ES, Banerjee S, Cunningham F, Harford WV, Kazi S, Little BB, Cryer B. The risk of acute myocardial infarction with etodolac in not increased compared to naproxen: A historical cohort analysis of a generic COX-2 selective inhibitor. J Cardiovasc Pharmacol Ther 2008; 13(4):252-260.
6. Velentgas P, West W, Cannuscio CC, Watson DJ, Walker AM. Cardiovascular risk of selective cyclooxygenase-2 inhibitors and non-aspirin non-steroidal anti-inflammatory medications. Pharmacoepidemiol Drug Saf 2006; 15:641-652.

**No data on individual NSAIDs**

1. Chan AT, Mason JE, Albert Cm, Chae CU, Rexrode KM, Curhan GC, Rimm EB, Willett WC, Fuchs CS. Non-steroidal anti-inflammatory drugs, acetaminophen, and the risk of cardiovascular events. Circulation 2006; 113:1578-87.
2. Hammad TA, Graham DJ, Staffa JA, Kornegay CJ, Dal Pan GJ. Onset of acute myocardial infarction after use of non-steroidal anti-inflammatory drugs. Pharmacoepidemiol Drug Saf 2008; 17:315-21.
3. Hawkey CJ, Hawkey GM, Everitt S, Skelly M, Stack WA, Gray D. Increased risk of myocardial infarction as first manifestation of ischemic heart disease and non-selective non-steroidal anti-inflammatory drugs. Br J Clin Pharmacol 2006; 61(6):730-7.
4. Mangonio AA, Woodman RJ, Gaganis P, Gilbert AL, Knight KM. Use of non-steroidal anti-inflammatory drugs and risk of incident myocardial infarction and heart failure, and all-cause mortality in the Australian veteran community. Br J Clin Pharmacol 2010; 69:689-700.
5. Pratt N, Roughead EE, Ryan P, Gilbert AL. Differential impact of NSAIDs on rate of adverse events that require hospitalization in high risk and general veteran populations. Drugs Aging 2010; 27(1):63-71.

**Channeling / usage pattern studies**

1. Fosbol El, Gislason GH, Jacobsen S, Abildstorm SZ, Hansen ML, Schramm TK, Folke F, Sorensen R, Rasmussen N,Kober L, Madsen M, Torp-Pedersen C. The pattern of use of non-steroidal anti-inflammatory drugs (NSAIDs) from 1997 to 2005: a nationwide study on 4.6 million people. Pharmacoepidemiol Drug Saf 2008; 17:822-33.
2. Depont F, fourrier A, Merliere Y, Droz C, Begaud B, Benichou J, Moride Y, Velo GP, Sturkenboom M, Blin P, More N. Channelling of COX-2 inhibitors to patients at higher gastrointestinal risk but not at lower cardiovascular risk: The COX-2 inhibitors and tNSAIDs description of users (CADEUS) study. Pharmacoepidemiol Drug Saf 2007; 16:891-900.
3. Layton D, Souverin PC, Heerdink ER, Shakir SAW, Egberts ACG. Evaluation of risk profiles for gastrointestinal and cardiovascular adverse effects in non-selective NSAID and COX-2 inhibitor users: A cohort study using pharmacy dispensing data in the Netherlands. Drug Saf 2008; 31(12):143-56.
4. Hsiao FY, Tsai YW, Huang WF. Changes in physicians’ practice of prescribing cyclooxygenase-2 inhibitor after market withdrawal of rofecoxib: A retrospective study of physician=patient pairs in Taiwan. Clin Ther 2009; 31(11):2618-27.

**Exposure not current /Unclear that it was current at time of cardiovascular event**

1. Jick H, Kaye JA, Russmann S, Jick SS. Non-steroidal anti-inflammatory drugs and acute myocardial infarction in patients with no major risk factors. Pharmacotherapy 2006; 26(10):1378-87.
2. Jick SS, Kaye JA, Jick H. Diclofenac and acute myocardial infarction in patients with no major risk factors. Br J Clin Pharmacol 2007; 64(5):662-7.
3. Turajane T, Wongbunnak R, Patcharatrakul T, Ratansumawong K, Poigampetch Y, Songpatanasilp T. Gastrointestinal and cardiovascular risk of non-selective NSAIDs and COX-2 inhibitors in elderly patients with knee osteoarthritis. J Med Assoc Thai 2009; 92(Suppl 6):S19-26.
4. Vaithiananathan R, Hockey PM, Moore TJ, Bates DW. Iatrogenic effects of COX-2 inhibitors in the US population: Findings from the medical expenditure panel survey. Drug Saf 2009; 32(4):335-43.

**Registry-based studies**

1. Nadareishvili Z, Michaud K, Hallenbeck JM, Wolfe F. Cardiovascualr, rheumatologic, and pharmacologic predictors of stroke in patients with rheumatoid arthritis: A nested case control study. ArthRheum 2008; 59(8):1090-6.
2. Goodson NJ, Brookhart AM, Symmons DP, Silman AJ, Solomon DH. Non-steroidal anti-inflammatory drug use does not appear to be associated with increased cardiovascular mortality in patients with inflammatory polyarthritis: results from a primary care based inception cohort of patients. Ann Rheum Dis 2009; 68:367-72.
3. Gudbjornsson B, Thorsteinsson SB, Sigvladason H, Einarsdottir R, Johannsson M, Zoega H, Halldorsson M, Thorgeirsson G. Rofecoxib, but not celecoxib, increases the risk of thromboembolic cardiovascular events in young adults: A nation-wide registry-based study. Eur J Clin Pharmacol 2010; 66:619-25.

**Duplicates**

1. Fosbol E, Andersson C, Weeke P, Olesen P, Kober L, Torp-Pederson C, Gislason GH. Use of non-steroidal anti-inflammatory drugs (NSAIDs) is associated with increased risk of stroke among healthy individuals. Eur Heart J 2010; 31(Abstract Supplement):619.
2. Gislason GH, Fosbol EL, Abildstrom SZ, Schramm TK, Hansen ML, Folke F, Kober L, Torp-Pedersen C. Exposure time and cardiovascular risk of NSAID treatment in patients with acute myocardial infarction or heart failure. Circulation 2008; 118:S_1130.
